# Preoperative sarcopenia combined with prognostic nutritional index predicts long-term prognosis of radical gastrectomy with advanced gastric cancer: a comprehensive analysis of two-center study

**DOI:** 10.1186/s12885-023-11251-0

**Published:** 2023-08-14

**Authors:** Yubo Han, Ju Wu, Rui Ji, Hao Tan, Simiao Tian, Jiajun Yin, Jian Xu, Xi Chen, Wenfei Liu, Hongzhang Cui

**Affiliations:** 1 Department of General Surgery, Affiliated Zhongshan Hospital of Dalian University, Dalian, China; 2 Fujian Medical University Union Hospital, Department of Gastric Surgery, Fuzhou, China; 3 Department of Medical Record and Statistics, Affiliated Zhongshan Hospital of Dalian University, Dalian, China; 4 Department of Radiology, Affiliated Zhongshan Hospital of Dalian University, Dalian, China

**Keywords:** Gastric cancer, Nutrition, Image, Sarcopenia, Prognosis, Combined index

## Abstract

**Purpose:**

This study aims to investigate the predictive value of the combined index smni(skeletal muscle index (SMI)-prognostic nutrition index(PNI)) for the postoperative survival of patients with advanced gastric cancer(AGC).

**Methods:**

650 patients with AGC from two centers (290 cases from the First Affiliated Hospital of Dalian University and 360 points from the Fujian Medical University Union Hospital) were selected as the study subjects based on unified screening criteria. Clinical data, preoperative abdominal CT images, results of hematology-related examinations, tumor-related characteristics, and surgical and follow-up data of the patients were collected and organized. The L3 vertebral level muscle area was measured using computer-assisted measurement techniques, and the skeletal muscle index(SMI) was calculated based on this measurement. The prognostic nutrition index (PNI) was calculated based on serum albumin and lymphocyte count indicators. The Kaplan-Meier survival analysis of data from the First Affiliated Hospital was used to determine that SMI and PNI are significantly correlated with the postoperative survival rate of patients with advanced gastric cancer. Based on this, a novel combined index smni was fitted and stratified for risk. Cox proportional hazards regression analysis was used to determine that the index smni is an independent prognostic risk factor for patients with AGC after surgery. The ROC curve was used to describe the predictive ability of the new combined index and its importance and predictive power in predicting postoperative survival of patients with AGC, which was verified in the data of Fujian Medical University Union Hospital.

**Result:**

The Kaplan-Meier curve analysis of the combined indicator smni Is clearly associated with long-term survival(3-year OS (P < 0.001) and DSS (P < 0.001)), univariate analysis and multivariate analysis showed that smni was an independent prognostic risk factor, The ROC curve for the first center 3-year OS(AUC = 0.678), DSS(AUC = 0.662) show good predictive ability and were validated in the second center.

**Conclusion:**

The combined index smni has a good predictive ability for the postoperative survival rate of patients with AGC and is expected to provide a new reference basis and more accurate and scientific guidance for the postoperative management and treatment of patients with AGC.

## Introduction

AGC is one of the most common cancers worldwide, with the highest incidence in East Asia [1]. Despite advances in medical technology and treatment methods, its survival rate remains relatively low [2]. Therefore, predicting the postoperative survival of patients with AGC effectively has become a hot topic of concern for clinical physicians and researchers. In recent years, the role of nutritional status in tumor prognosis has received increasing attention [3, 4]. The Prognostic nutritional index (PNI) is an indicator that comprehensively evaluates the nutritional status of patients [5, 6], which has been widely used to predict the survival of cancer patients. However, the single application of the PNI index has certain limitations because it cannot reflect the biological characteristics of the tumor itself.

Skeletal muscle index (SMI) assesses the patient’s muscle mass by calculating the ratio of muscle area to body mass, which is a relatively simple, non-invasive, and economical method of preoperative nutritional assessment and is significantly correlated with the prognosis of multiple cancers [7]. To improve the prediction of the postoperative survival of patients with AGC, this study will develop a novel combined index smni (preoperative SMI combined with PNI), using a dual-center validation method.

**Fig. 1:**
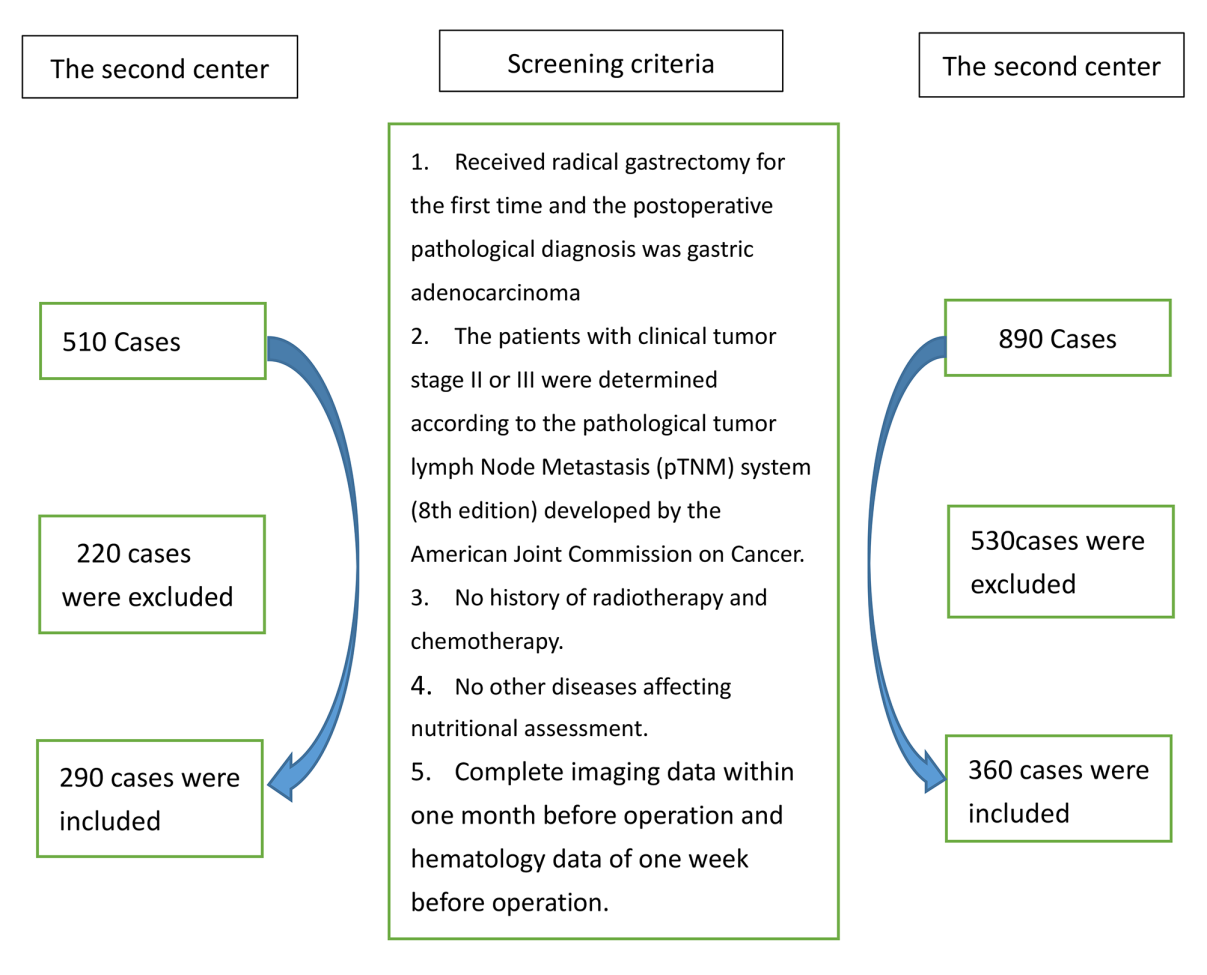
Deletion criteria for inclusion in the study

## Materials and methods

### Collection and processing of data

This study included patients diagnosed with AGC in two centers who underwent radical surgery between January 2011 and December 2019. The selection criteria were: (1) Received radical gastrectomy for the first time and the postoperative pathological diagnosis was gastric adenocarcinoma; (2) The patients with clinical tumor stage II or III were determined according to the 8th American Joint Committee on Cancer (AJCC) staging system(pTNM staging); (3) No history of radiotherapy and chemotherapy; (4) No other diseases affecting nutritional assessment; (5) Complete imaging data within one month before operation and hematology data of one week before the operation; (6) Patients receiving conventional chemotherapy after surgery. A total of 650 patients who met the selection criteria were included in this study (as shown in Fig. 1). We analyzed the demographic, histopathological, and laboratory data of the study subjects, extracted relevant information from patient records and hospital databases, and determined the tumor stage according to the pathological tumor lymph node metastasis (pTNM staging) system (8th edition) formulated by the American Joint Committee on Cancer. Postoperative follow-up was conducted every three months during the first two years and every six months after two years. The last follow-up date was December 31, 2022. Overall survival (OS) was defined as the duration between the date of surgery and the date of the final follow-up or death and was used as the primary endpoint[8]. DSS is defined as the time between the date of operation and the date of all-cause death as the secondary endpoint [9].

In this study, 290 patients were from the First Center of Department of General Surgery, Affiliated Zhongshan Hospital of Dalian University, while 360 patients were from the Second Center of Department of Gastric Surgery, Fujian Medical University Union Hospital. According to the recent guidelines of the European Working Group on Sarcopenia in Older People (EWGSOP), sarcopenia is defined as a combination of low muscle mass plus either low grip strength or slow gait speed [10]. The role of low muscle strength outweighs that of low muscle mass as the primary determinant of sarcopenia [10]. Our study design was retrospective and unable to collect information on muscle function (muscle strength or physical performance). Therefore, we only evaluated muscle mass to determine whether patients had sarcopenia [8].

### Acquisition and processing of imaging data

**Fig. 2:**
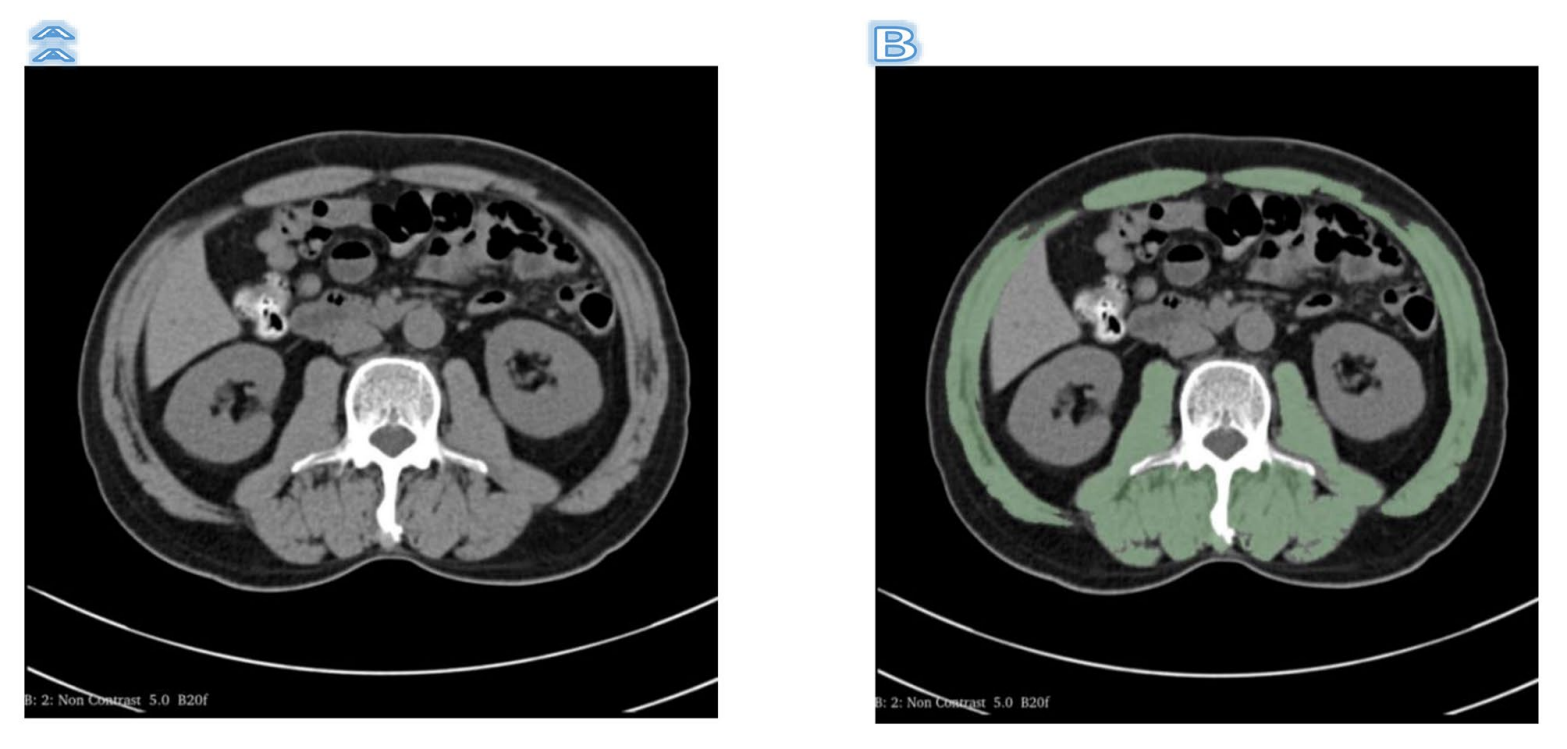
**(A)** The height scan image of the L3 cone without outline; **(B)** The height scan image of the L3 cone after outline, the green area is the outline part

CT-related parameters were as follows: routine section thickness 5.0 mm. These 650 patients’ chest CT data were procured from the picture archiving and communication system (PACS). The software 3DSlicer [11]was used to identify and quantify muscle tissue [12], which allows for selective visualization of specific tissues, such as muscle or fat tissue, by setting density intervals representing the tissue of interest. According to the current literature, a density range of -29 to + 150 Hounsfield units (HU) was selected. The skeletal muscle tissue was then segmented in more detail at the L3 level, where the transverse processes of the vertebrae were visible on the L3 plane. We drew a region of interest (ROI) corresponding to the total lumbar muscle area (TLA), which includes lumbar, paraspinal, and abdominal wall muscles, and the size (cm2) was automatically calculated using the software as shown in Fig. 2.

The SMI was calculated for each patient, a quantitative parameter obtained from the ratio of TLA to height squared (cm2/m2). This index represents the standardized index of skeletal muscle mass relative to the patient’s size.

$$SMI = \frac{{TLA}}{{Hight{\text{ }}Squared}}$$

Using X-TILE[13]to determine the cut-off value, a score of 0 was assigned to patients with SMI < 39.5 cm2/m2 for men and SMI < 29.5 cm2/m2 for women, indicating sarcopenia and a score of 1 was assigned to patients who did not have sarcopenia. A radiologist from Zhongshan Hospital, Dalian University, performed the reconstruction and acquisition of CT parameters consistently in both centers.

### Acquisition and processing of the hematological data

The PNI was calculated as follows: serum albumin concentration (g/dL) + 5 × peripheral lymphocyte count (number/mm2).

$$\text{P}\text{N}\text{I}=\text{A}\text{L}\text{B}+5\times \text{L}\text{Y}\text{M}$$

This study calculated PNI using preoperative whole blood count and serum albumin examination data from the previous month. X-TILE was used to determine the cutoff value for PNI as 46, where PNI > 46 was assigned a score of 1 for the high PNI group and 0 for the low PNI group. This study’s main focus was to observe patients’ survival status, with a minimum follow-up time of 3 years. In Kaplan-Meier survival analysis, both SMI and PNI were significantly correlated with 3-year DSS and 3-year OS, and there was no collinearity issue between these two indicators (collinearity diagnosis VIF = 1). Based on these findings, a novel combined index was established and named smni, and its risk stratification was performed, i.e., high-risk group: combined index smni = 0, i.e., SMI = 0 and PNI = 0; medium-risk group: combined index smni = 1, i.e., SMI = 0 and PNI = 1 or SMI = 1 and PNI = 0; low-risk group: combined index smni = 2, i.e., SMI = 1 and PNI = 1. Kaplan-Meier curves were plotted to clarify their significant correlation with patients’ 3-year DSS (disease-specific survival) and 3-year OS (overall survival). In addition, Cox proportional hazards regression analysis validated that the combined index smni was an independent prognostic factor for gastric cancer patients. Receiver Operating Characteristic(ROC) curves were used to verify the predictive ability of the new combined index smni in terms of prognosis. Furthermore, in the second center’s gastric cancer database, with 3-year OS as the endpoint event, the same set of research factors was used to establish the same criteria for the combined index smni, aiming to verify the general applicability of the combined index. Kaplan-Meier curves, Cox proportional hazards regression analysis, and ROC curve analysis were used to demonstrate the predictive ability of the new combined index.

### Data statistics

The data were statistically analyzed using SPSS 27.0 (SPSS Inc., Chicago, IL, USA) and R 4.2.2. P values were calculated using the log-rank test for Kaplan-Meier survival analysis. Cox proportional hazard analysis results were expressed as hazard ratios (HR), which indicate the relative risk between two groups, i.e., the multiple survival risk of patients in one group compared to those in the control group. An HR value greater than 1 indicates a higher survival risk for patients in that group, while an HR value less than 1 indicates a lower survival risk. The HR value’s confidence interval (CI) is also an important indicator of Cox joint index analysis, with a narrower CI indicating more reliable results. The area under the curve (AUC) of the ROC curve was used to compare the predictive abilities of different indicators.

## Results

This study enrolled 290 eligible patients who received treatment at the first center. The average age of the patients was 66.06 years, with 212 males and 88 females. There were 76 males and 33 females in the sarcopenia group and 136 males and 45 females in the non-sarcopenia group. The two groups showed significant statistical differences in age (p = 0.006), BMI (p = 0.042), hematological index FIB (p = 0.006), postoperative pathological pTNM Staging (p = 0.001), 3-year overall survival (OS) (p < 0.001), and disease-specific survival (DSS) (p < 0.001). The two groups had no significant statistical differences regarding gender, alcohol consumption, tumor marker CEA, hematological index LDH (lactate dehydrogenase), and tumor size. The high PNI group (154 males and 63 females) and the low PNI group (58 males and 15 females) showed significant differences in BMI (p = 0.97), tumor marker CEA (P = 0.022,), and tumor size (P = 0.009, but not in postoperative pathological pTNM Staging (P = 0.086). There were significant differences in patients’ 3-year OS (P = 0.017) and 3-year DSS (P = 0.04) as shown in Table 1,

**Fig. 3:**
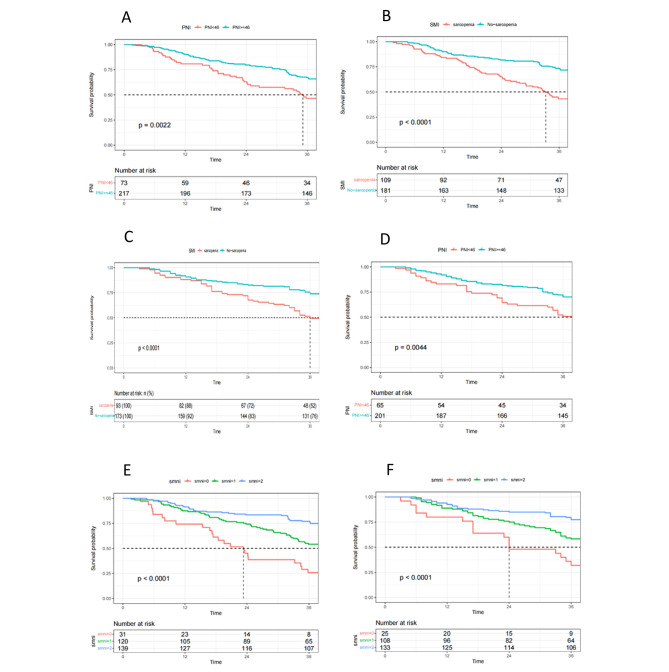
3-year OS, DSS survival curves for PNI, SMI, and smni. **(A), (B)**, (E)3-year OS survival curve of SMI, PNI, smni; **(C)(D)(F)** 3-year DSS survival curve for the first center SMI, PNI, smni

and the second center correlation analysis is shown in Table 2. The Kaplan-Meier curves for 3-year OS and DSS for sarcopenia and non-sarcopenia patients and high PNI and low PNI groups were shown in Fig. 3**(A)-(F)**, respectively. The logarithmic rank test also confirmed significant statistical differences between the two groups. The Kaplan-Meier curve analysis of the combined indicator smni Is clearly associated with long-term survival(3-year OS (P < 0.001) and DSS (P < 0.001)). In the Univariate Analysis, Fib (p < 0.001, Hr = 2.241), CEA (p < 0.001, Hr = 2.029), Size (P < 0.001, HR = 0.529), LDH (P = 0.032, HR = 0.529), and pTNM staging (P = 0.001, HR = 5.602) were shown to have statistical significance. The combined indicator smni (moderate risk group P = 0.001, HR = 0.438; low-risk group P < 0.01, HR = 0.224) was also shown to have statistical significance as a prognostic factor for OS. When these factors were included in the multivariate analysis, only Fib (p = 0.016, Hr = 1.655), CEA (p = 0.002 h = 1.905), pTNM staging (P = 0.001, HR = 4.215), and the combined indicator smni (moderate risk group P = 0.004, HR = 0.494; low-risk group P = 0.001, HR = 0.374) were shown to be independent adverse predictors of 3-year OS, as shown in Table 3.

**Table 1:** Statistical information of different groups in the study in the first Center

Variable	Sarcopenia (N = 109)	Non-sarcopenia (N = 181)	*P*-value	PNI ≤ 46 (N = 73)	PNI > 46 (N = 217)	*P*-value
**Age**			0.006			0.968
≤ 60	18 (16.5%)	58 (32.0%)		19 (26.0%)	57 (26.3%)	
> 60	91 (83.5%)	123 (68.0%)		54 (74.0%)	160(73.7%)	
**Sex**			0.31			0.207
Male	76 (69.7%)	136 (75.1%)		58 (79.5%)	154(71.0%)	
Female	33 (30.3%)	45 (24.9%)		15 (20.5%)	63 (29.0%)	
**Smoke**			0.808			0.208
No	71 (65.1%)	114 (63.0%)		51 (69.9%)	134(61.8%)	
Yes	38 (34.9%)	67 (37.0%)		22 (30.1%)	83 (38.2%)	
**Drink**			0.385			0.102
No	83 (76.1%)	128 (70.7%)		59 (80.8%)	152(70.0%)	
Yes	26 (23.9%)	53 (29.3%)		14 (19.2%)	65 (30.0%)	
**BMI**			0.042			0.97
< 25	89 (81.7%)	127 (70.2%)		54 (74.0%)	162(74.7%)	
≥ 25	20 (18.3%)	54 (29.8%)		19 (26.0%)	55 (25.3%)	
**Fib**			0.006			0.267
≤ 3.58	63 (57.8%)	133 (73.5%)		45 (61.6%)	151(69.6%)	
> 3.58	46 (42.2%)	48 (26.5%)		28 (38.4%)	66 (30.4%)	
**CEA**			0.368			0.022
≤ 4.2	61 (56.0%)	111 (61.3%)		35 (47.9%)	137(63.1%)	
> 4.2	48 (44.0%)	70 (38.7%)		38 (52.1%)	80 (36.9%)	
**LDH**			0.900			0.906
≤ 179	24(22.0%)	41(22.6%)		16(21.9%)	49(22.5%)	
> 179	85(78.0%)	140(77.4%)		57(78.1%)	168(77.5%)	
**Size**			0.425			0.009
<=4	58 (53.2%)	105 (58.0%)		31 (42.5%)	132(60.8%)	
> 4	51 (46.8%)	76 (42.0%)		42 (57.5%)	85 (39.2%)	
**pTNM**			0.001			0.086
II	27(24.8%)	87 (48.1%)		22(30.1%)	92(42.4%)	
III	82 (75.2%)	94 (51.9%)		51 (69.9%)	125(57.6%)	
**OS**			< 0.001			0.017
Alive	40 (36.7%)	122 (67.4%)		32(43.8%)	130(59.9%)	
Dead	69 (63.3%)	59 (32.6%)		41 (56.2%)	87 (40.1%)	
**DSS**			< 0.001			< 0.001
Alive	56(60.2%)	138(79.7%)		Alive	41(63.1%)	
Dead	37(39.8%)	35(21.3%)		Dead	24(36.9%)	

**Table 2:** Statistical information of different groups in the study in the second center

	Sarcopenia	Non-sarcopenia	*P*-value	PNI ≤ 46	PNI > 46	*P*-value
**Age**			< 0.001			0.002
≤ 60	52 (38.8%)	155 (68.9%)		106 (50.7%)	101 (67.3%)	
> 60	82 (61.2%)	70 (31.1%)		103 (49.3%)	49 (32.7%)	
**Sex**			0.318			0.496
Male	94 (70.1%)	170 (75.6%)		157 (75.1%)	107 (71.3%)	
Female	40 (29.9%)	55 (24.4%)		52 (24.9%)	43 (28.7%)	
**Smoke**			0.184			0.456
No	94 (70.1%)	141 (62.7%)		133 (63.6%)	102 (68.0%)	
Yes	40 (29.9%)	84 (37.3%)		76 (36.4%)	48 (32.0%)	
**Drink**			0.751			0.146
No	96 (71.6%)	166 (73.8%)		146 (69.9%)	116 (77.3%)	
Yes	38 (28.4%)	59 (26.2%)		63 (30.1%)	34 (22.7%)	
**BMI**			0.002			0.516
< 25	128(95.5%)	189(84%)		187(89.4%)	130(86.6%)	
≥ 25	6(4.5%)	36(16%)		22(10.6%)	20(13.4%)	
**FIb**			0.262			0.813
≤ 3.58	53 (39.6%)	104 (46.2%)		93 (44.5%)	64 (42.7%)	
> 3.58	81 (60.4%)	121 (53.8%)		116 (55.5%)	86 (57.3%)	
**LDH**			0.144			0.634
≤ 179	28 (20.9%)	64 (28.4%)		56 (26.8%)	36 (24.0%)	
> 179	106 (79.1%)	161 (71.6%)		153 (73.2%)	114 (76.0%)	
**CEA**			0.09			0.641
≤ 4.2	81 (60.4%)	157 (69.8%)		136 (65.1%)	102 (68.0%)	
> 4.2	53 (39.6%)	68 (30.2%)		73 (34.9%)	48 (32.0%)	
**pTNM**			0.219			0.357
II	35 (26.1%)	74 (32.9%)		59 (28.2%)	50 (33.3%)	
III	99 (73.9%)	151 (67.1%)		150 (71.8%)	100 (66.7%)	
**Size**			0.157			0.407
<=4	79 (59.0%)	114 (50.7%)		108 (51.7%)	85 (56.7%)	
> 4	55 (41.0%)	111 (49.3%)		101 (48.3%)	65 (43.3%)	
**OS**			< 0.001			< 0.001
Alive	44 (32.8%)	131 (58.2%)		82 (39.2%)	93 (62.0%)	
Dead	90 (67.2%)	94 (41.8%)		127 (60.8%)	57 (38.0%)	

**Table 3:** Univariate and multivariate Cox regression analysis for overall survival

variable	Univariate Analysis			Multivariate Analysis				
	*P*-value	HR	95.0% CI		*P*-value	HR	95.0% CI	
Sex	0.095	0.695	0.454–1.065					
Age	0.571	1.126	0.747–1.697					
Smoke	0.396	1.168	0.816–1.673					
Drink	0.96	0.99	0.668–1.467					
BMI	0.528	0.877	0.584–1.317					
Fib	< 0.001	2.241	1.576–3.188		0.003	1.743	1.214–2.501	
CEA	< 0.001	2.029	1.429–2.879		0.01	1.599	1.12–2.283	
LDH	0.032	0.599	0.375–0.957					
Size	< 0.001	0.529	0.372–0.75					
pTNM(II)								
pTNM(III)	< 0.001	5.602	3.397–9.239		0.001	4.215	2.518–7.056	
smni(0)								
smni(1)	0.001	0.438	0.272–0.706		0.004	0.494	0.306–0.797	
smni(2)	< 0.001	0.224	0.135–0.373		0.001	0.376	0.223–0.632	

**Fig. 4:**
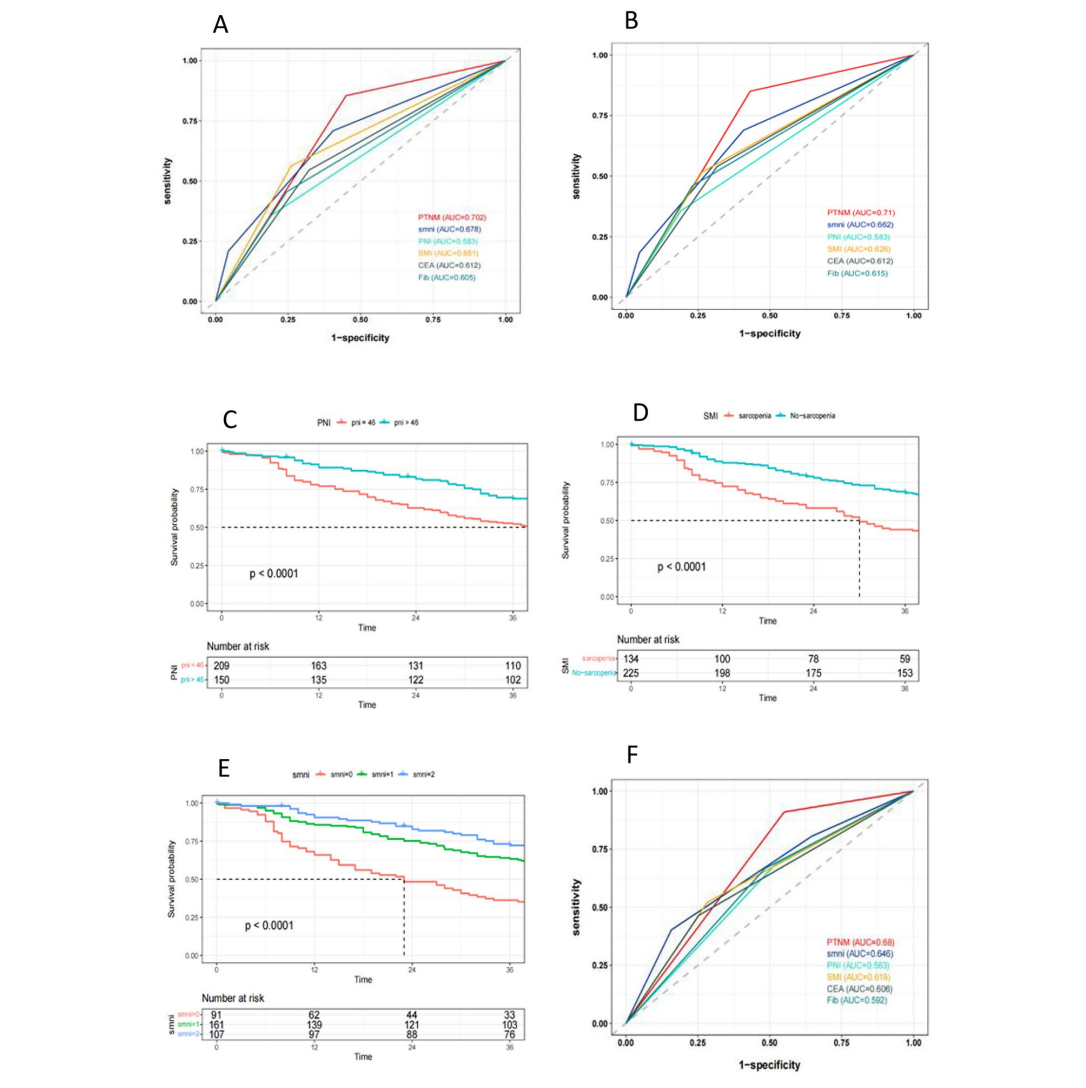
The ROC curve for the first center 3-year OS, DSS and the second center PNI, SMI, smni, and the ROC curve for each indicator in the 3-year OS; **(A)** The ROC curve analysis of each indicator in the 3-year OS of the first center. **(B)** The ROC curve analysis of each indicator in the 3-year DSS of the first center. **(C)(D)(E)** KM curve analysis of the second central PNI, SMI, smni, **(F)** The ROC curve analysis of each indicator in the 3-year OS of the second center

Regarding the analysis of the 3-year disease-specific survival (DSS) of patients, the results of univariate regression analysis showed that Fib (p < 0.001, HR = 2.21), CEA (p < 0.001, HR = 2.166), tumor size (p = 0.003, HR = 1.81), pTNM staging (P < 0.001, HR = 4.831), and the combined index smni (intermediate-risk group P = 0.005, HR = 0.456; low-risk group P < 0.001, HR = 0.238) were significant prognostic factors for DSS. The results of multivariate analysis showed that Fib (p < 0.016, HR = 1.655), CEA (p = 0.002, HR = 1.905), pTNM staging (P = 0.001, HR = 4.831), and the combined index smni (intermediate-risk group P = 0.015, HR = 0.508; low-risk group P = 0.002, HR = 0.397) were independent predictors of poor prognosis for DSS, as shown in Table 4. According to the independent prognostic factors index of COX proportional hazard regression analysis, ROC curves were drawn to predict the overall survival (OS) of the first center at three years (as shown in Fig. 4**(A)**). The area under the curves (AUC) of each index were as follows: combined index smni: AUC = 0.678, Fib: AUC = 0.605, CEA: AUC = 0.612, SMI: AUC = 0.651, PNI: AUC = 0.563, pTNM staging: AUC = 0.702. To predict the survival of 3-year DSS (as shown in Fig. 4**(B)**), the AUC of each index were as follows: combined index smni: AUC = 0.662, Fib: AUC = 0.615, CEA: AUC = 0.612, SMI: AUC = 0.626, PNI: AUC = 0.583, pTNM staging: AUC = 0.71. Similarly, our analysis of 3-year DSS in gastric cancer patients also showed the excellent predictive ability of the combined index smni. The verification results of 3- year OS in the second center are shown in Fig. 4**(C)-(F)**.

**Table 4:** Univariate and multivariate Cox regression analysis for disease-specific survival

variable	Univariate Analysis				Multivariate Analysis		
	*P*-value	HR	95.0% CI		*P*-value	HR	95.0% CI
Sex	0.392	0.823	0.527–1.285				
Age	0.925	0.979	0.634–1.513				
Smoke	0.586	1.118	0.749–1.667				
Drink	0.488	0.851	0.538–1.344				
BMI	0.528	0.877	0.584–1.317				
Fib	< 0.001	0.436	0.295–0.645		0.004	1.799	1.204–2.69
CEA	0.049	0.593	0.352–0.999		0.016	1.628	1.096–2.42
LDH	< 0.001	2.094	1.419–3.09				
Size	0.002	1.866	1.267–2.75				
pTNM(II)							
pTNM(III)	< 0.001	4.831	2.867–8.138		0.001	4.096	2.359–7.111
smni(0)							
smni(1)	0.005	0.456	0.265–0.786		0.015	0.508	0.294–0.877
smni(2)	< 0.001	0.238	0.134–0.422		0.002	0.397	0.222–0.713

## Discussion

Various indicators have been proposed to predict the postoperative survival of gastric cancer patients, such as tumor size, lymph node metastasis, hematological indicators, etc. [14–17]. However, these indicators have different limitations and cannot fully reflect the prognosis of gastric cancer patients. Although surgical resection is the mainstay of cancer treatment, the prediction of radical gastrectomy is related to the changes in patients’ postoperative metabolism, endocrine, neuroendocrine, and immune systems, affecting the patient’s postoperative survival and quality of life. The combined index smni proposed in this study significantly correlates with the long-term prognosis of AGC after surgery. It is based on the combination of nutrition-related PNI and SMI. Patients with a high PNI and SMI have relatively good nutritional status, and their long-term survival rate is higher. The SMI value at the L3 level defines whether GC patients have sarcopenia. In different studies, the cutoff value of SMI varies. It is 36 to 53 cm2/m2 for men, and for women, it is 29 to 41 cm2/m2. The cutoff value used in this study is also within this range. Several studies on different types of tumors (colorectal, liver, pancreas) have shown that low SMI is an independent prognostic factor for survival[18–22]. Sarcopenia is associated with increased hospitalization time, postoperative complications, mortality, and decreased survival [23–25]. The reason may be that muscle factors produced by skeletal muscles may have anti-inflammatory and anticancer effects. In contrast, highly invasive tumors often have higher metabolic activity, leading to nutritional consumption and decreased muscle cells. Therefore, the loss of muscle tissue leads to reduced secretion of muscle factors [26].

Of course, many other methods have been proven effective and feasible for assessing sarcopenia, such as grip strength testing. It can serve as a reliable substitute indicator for more complex arm and leg strength measurements. Low grip strength is a vital predictive factor for poor prognosis in patients, including prolonged hospital stay, increased costs, poor quality of life, and elevated mortality rates [27]. Studies have also shown that the grip strength in patients undergoing tumor surgery can directly reflect the nutritional status and long-term survival and not affected by the age of patients [28], there are also studies showing that after total gastric resection, nutrition affect the quality of patient’s life, we can through monitoring and timely intervention to improve the postoperative quality of life [29]. Since our study is retrospective and limited by the evaluation conditions of previous patients, we used imaging techniques to identify the sarcopenic status of patients. This method has been validated in several previous studies [30, 31] .

When mentioning grip strength in relation to nutritional analysis, it inevitably raises concerns about the frailty. The syndrome of frailty is associated with, but not an inevitable consequence of ageing and is characterized by a vulnerability to stressor events that can be both internal and external.6 Both frailty and pre-frailty, the prodromal state before the onset of clinically identifiable frailty, are associated with adverse outcomes [32]. The most widely used definitions of physical frailty are the phenotype model described by Fried, where frailty is identified by the presence of at least three out of five physical characteristics: weight loss, exhaustion, low energy expenditure, slow walking speed, and low handgrip strength [33]. The cumulative deficit model of frailty described by Rockwood et al. also predicts adverse health outcomes and comprises age-associated accumulation of deficits that range from symptoms, sensory deficits, clinical signs, diseases, disabilities, and abnormal laboratory test results [34], Because our analysis is retrospective, we do not have a complete collection of some indicators related to patient supervisor feelings,We will make up for the data in the future work and This will be an expansion direction of our future clinical research。.

Body mass index (BMI) is a widely used and helpful indicator of nutritional status, but its impact on treatment outcomes in gastric cancer (GC) patients remains controversial [35–37]. In our study, BMI showed a significant correlation with sarcopenia. Still, it did not have a particular significance in predicting long-term survival, possibly because normal and high BMI can mask the actual nutritional status of patients.

Whether patients receive chemotherapy or neoadjuvant chemotherapy also affects their nutritional status. Low tolerance to chemotherapy can develop sarcopenia and impact patients’ long-term survival [38], as suggested by previous studies[39]. There is a statistically significant difference in survival rates between sarcopenic and non-sarcopenic patients after adjuvant chemotherapy in an analysis of esophagogastric tumor patients, and changes in body composition following neoadjuvant chemotherapy showed a significant increase in the number of patients with muscle loss [40]. Therefore, we excluded patients who did not receive regular chemotherapy after surgery, and we chose not to analyze the effect of neoadjuvant chemotherapy on sarcopenic patients because neoadjuvant therapy alters the tumor’s original pathological staging, which is beyond the scope of our study and also included in our exclusion criteria. We hope that future scholars can conduct more in-depth investigations in this regard.

However, the response of muscle loss to nutritional status has a certain lag and is less sensitive than blood indicators to detect and promptly reflect the patient’s state. Therefore, PNI is included to improve the prediction system.

Previous research has shown that PNI is a new systemic immune-nutritional index that represents the immune and nutritional status of the host and is an essential biomarker for various tumors [41, 42]. Initially, PNI was developed to evaluate the incidence of postoperative complications in patients undergoing gastrointestinal surgery[43]. Subsequently, more studies found that PNI is closely related to the long-term prognosis of tumors and is an independent prognostic factor for the survival of various cancer patients [41, 42, 44, 45]. PNI is calculated using serum albumin and peripheral blood lymphocytes, which represent the nutritional and immune status of the body, respectively.

Lymphocytes play a crucial role in tumor-related immunology. They possess potent anti-tumor immune functions and can inhibit the progression of various malignancies [46]. Elevated levels of lymphocytes have been reported to correlate with favorable prognosis in multiple types of tumors [47]. Additionally, several subtypes of tumor-infiltrating lymphocytes have been associated with improved prognosis in various malignancies [47], including CD8 + T cells [19] and memory T cells [48]. However, specific subsets of T cells have been associated with tumor progression and poor prognoses, such as regulatory T cells and Th17 cells [49]. Despite the association of different T cell subsets with adverse tumor prognosis, in our study, high absolute lymphocyte count levels correlate with a favorable prognosis in gastric cancer patients.

In recent years, several lymphocyte-based inflammatory markers, including NLR, LMR, and PLR, have been reported as indicators of treatment response in cancer patients. High NLR and PLR, as well as low LMR, are considered markers of nonspecific immune system activation and are significantly associated with adverse clinical outcomes in various cancers, including AGC patients[50], small cell lung cancer [51], colorectal cancer [52], pancreatic cancer [53], and non-small cell lung cancer [54].

Recent evidence has confirmed that lower serum albumin levels predict higher mortality rates, particularly regarding prognosis among cancer patients, such as colon cancer[55]. There have also been studies investigating the association of serum albumin with other ratios, including albumin-to-globulin ratio, C-reactive protein-to-albumin ratio, albumin-to-alkaline phosphatase ratio, and albumin-to-fibrinogen ratio [55, 56]. These studies have also demonstrated an association with cancer prognosis, potentially linked to inflammatory responses. Furthermore, albumin is considered an essential extracellular antioxidant. Animal experiments have shown that serum albumin can act as an antioxidant, reducing arterial reactivity induced by endotoxemia and enhancing anti-inflammatory effects. However, further research is needed to elucidate the precise mechanisms by which albumin impacts cancer prognosis in humans.

Nutrition-related indicators also include Prealbumin and retinol-binding proteins, and research has shown that these markers are significantly associated with long-term survival in cancer patients[57–60]. Prealbumin concentration is closely related to early changes in nutritional status. It can detect early malnutrition[61], indicating its sensitivity in assessing patients’ nutritional quality in the early postoperative period. Furthermore, prealbumin is less affected by age and inflammation [62], making it a more stable and reliable predictive indicator for cancer prognosis. Therefore, researchers have used it with notable effectiveness to predict the prognosis of various malignancies, such as lung cancer, esophageal cancer, and renal cell carcinoma [58–60]. However, these indicators are beyond the scope of our current study and only serve as additional research directions for further discussion.

Studies have shown [63] that malnutrition is widespread in cancer patients, with an incidence ranging from 39 to 71%, and is always accompanied by a higher incidence of infections, treatment-related toxicity, and subsequent adverse reactions that ultimately lead to more extended hospital stays. The immune system represents the body’s ability to monitor and clear external invaders (such as external viruses) and mutated internal cells (such as cancer cells) [64, 65]. Malnutrition or poor immune status often leads to a significant decrease in the body’s defense capabilities, leading to local recurrence or distant metastasis of tumors, ultimately shortening the progression-free survival (PFS), distant metastasis-free survival (DMFS), and overall survival (OS) of patients[66]. Our center’s research also found a significant correlation between low preoperative PNI and poor long-term survival after gastric cancer surgery.

As mentioned above, both SMI and PNI have been studied in malignant tumors. Still, we are the first to use them in combination to predict the postoperative survival of gastric cancer patients. The results show that the combined indicator smni is significantly correlated with OS and DSS of postoperative gastric cancer patients. In the data from two centers, the predictive ability of the combined indicator smni for the postoperative survival of gastric cancer patients as an independent risk factor is similar to and significantly higher than that of other risk factors such as FIB and CEA (which can be supported by relevant literature). It is also higher than that of SMI and PNI used for fitting. This suggests that the combined indicator smni has a more excellent and stable predictive ability, and may assist in treating postoperative patients.

However, this study also has some limitations. Firstly, although a dual-center verification method was used to reduce bias, there is still some information bias and patient selection bias. Secondly, further research is needed to verify its effectiveness and reliability in a larger sample and more diverse population. At last, we excluded patients who received neoadjuvant chemotherapy prior to surgery. Future studies can also explore other aspects of the issue, such as the relationship between the combined indicator smni and other predictors of postoperative survival in gastric cancer patients, such as inflammatory markers, biochemical blood indicators, and other nutritional indicators, to determine the advantages and scope of application of the combined indicator smni in clinical practice. In addition, the combined indicator smni can be combined with other factors that affect a patient’s prognosis to establish a more comprehensive predictive indicator to improve the accuracy of prediction and guide patients’ treatment and care.

## Data Availability

The data set or related content involved in this manuscript can be obtained from the first author or the corresponding author.
